# Digitalization and disruptive change in rheumatology

**DOI:** 10.1007/s00393-022-01222-4

**Published:** 2022-05-31

**Authors:** Pia Richter, Jutta G. Richter, Elke Lieb, Friedrich Steimann, Gamal Chehab, Arnd Becker, Christian Thielscher

**Affiliations:** 1 Competence Center for Medical Economics, FOM University, Sigsfeldstr. 5, 45141 Essen, Germany; 2 Policlinic for Rheumatology and Hiller Research Unit for Rheumatology, Medical Faculty, Heinrich-Heine-University Duesseldorf, University Clinic, Moorenstr. 5, 40225 Duesseldorf, Germany; 3 FOM University, Am Kieselhumes 15, 66123 Saarbrücken, Germany; 4 Department for Programming Systems, FernUniversität Hagen, Universitätsstraße 11, 58097 Hagen, Germany; 5 Ortenau Klinikum Offenburg-Kehl, Offenburg, Germany

**Keywords:** Rheumatology, Information and communication technology, Patient-doctor relationship, Medical profession, Organization of treatment, Rheumatologie, Informations- und Kommunikationstechnologie, Arzt-Patienten-Beziehung, Ärztlicher Beruf, Behandlungsorganisation

## Abstract

**Introduction:**

Recently, many sectors have seen disruptive changes due to the rapid progress in information and communication technology (ICT). The aim of this systematic literature review was to develop a first understanding of what is known about new ICTs in rheumatology and their disruptive potential.

**Methods:**

PubMed, LIVIVO, and EBSCO Discovery Service (EDS) databases were searched for relevant literature. Use of new ICTs was identified, categorized, and disruptive potential was discussed. Articles from 2008 to 2021 in German and English were considered.

**Results:**

A total of 3539 articles were identified. After application of inclusion/exclusion criteria, 55 articles were included in the analyses. The majority of articles (48) used a non-experimental design or detailed expert opinion. The new ICTs mentioned in these articles could be allocated to four main categories: technologies that prepare for the development of new knowledge by data collection (*n* = 32); technologies that develop new knowledge by evaluation of data (e.g., by inventing better treatment; *n* = 11); technologies that improve communication of existing knowledge (*n* = 32); and technologies that improve the care process (*n* = 29). Further assessment classified the ICTs into different functional subcategories. Based on these categories it is possible to estimate the disruptive potential of new ICTs.

**Conclusion:**

ICTs are becoming increasingly important in rheumatology and may impact patients’ lives and professional conduct. The properties and disruptive potential of technologies identified in the articles differ widely. When looking into ICTs, doctors have focused on new diagnostic and therapeutic procedures but rarely on their disruptive potential. We recommend putting more effort into investigation of whether ICTs change the way rheumatology is performed and who is in control of it. Especially technologies that potentially replace physicians with machines, take control over the definition of quality in medicine, and/or create proprietary knowledge that is not accessible for doctors need more research.

**Supplementary Information:**

The online version of this article (10.1007/s00393-022-01222-4) includes Table S1.

## Abstract (de)

**Hintergrund:**

In jüngster Zeit haben sich in vielen Branchen aufgrund des raschen Fortschritts in der Informations- und Kommunikationstechnologie (IKT) disruptive Veränderungen ereignet. Ziel dieser systematischen Literaturrecherche war es, ein erstes Verständnis dafür zu entwickeln, was über neue IKT in der Rheumatologie und ihr disruptives Potenzial bekannt ist.

**Methoden:**

Die Datenbanken PubMed, LIVIVO und EBSCO Discovery Service (EDS) wurden nach relevanter Literatur durchsucht. Der Einsatz neuer IKT-Technologien wurde identifiziert, kategorisiert und das disruptive Potenzial diskutiert. Dabei wurden Artikel aus den Jahren 2008 bis 2021 in deutscher und englischer Sprache berücksichtigt.

**Ergebnisse:**

Es wurden 3539 Artikel identifiziert; nach Anwendung von Einschluss- und Ausschlusskriterien wurden 55 Artikel in die Analyse aufgenommen. Die Mehrheit der Artikel (48) verwendete ein nichtexperimentelles Design oder stützte sich auf Expertenmeinungen. Die in diesen Artikeln erwähnten neuen IKT ließen sich 4 Hauptkategorien zuordnen: Technologien, die auf die Entwicklung von neuen Erkenntnissen durch Datenerhebung zielen (*n* = 32); Technologien, die neues Wissen durch die Auswertung von existierenden Daten entwickeln (z. B. durch das Auffinden besserer Behandlungsmöglichkeiten) (*n* = 11); Technologien, die die Vermittlung von vorhandenem Wissen verbessern (*n* = 32); und Technologien, die den Versorgungsprozess verbessern (*n* = 29). Bei der weiteren Bewertung der IKT konnten diese in verschiedene funktionale Unterkategorien eingeteilt werden; anhand dieser Kategorien lässt sich das disruptive Potenzial neuer IKT abschätzen.

**Schlussfolgerung:**

IKT gewinnen in der Rheumatologie zunehmend an Bedeutung und können das Leben der Patienten und die ärztliche Berufsausübung beeinflussen. Sowohl die Eigenschaften als auch das disruptive Potenzial der in den Artikeln genannten Technologien sind sehr unterschiedlich. Bei der Untersuchung der IKT haben sich Ärzte bisher auf neue diagnostische und therapeutische Verfahren konzentriert, aber selten auf ihr disruptives Potenzial. Die Autoren empfehlen, mehr Anstrengungen zu unternehmen, um zu untersuchen, ob IKT die Art und Weise, wie Rheumatologie durchgeführt wird, verändern und wer die Kontrolle darüber hat. Insbesondere Technologien, die potenziell Ärzte durch Maschinen ersetzen, die Kontrolle über die Definition von Qualität in der Medizin übernehmen und/oder proprietäres Wissen schaffen, das Ärzten nicht zugänglich ist, bedürfen weiterer Forschung.

## Introduction

Recently, many sectors have seen massive changes—often labeled “disruptive”—due to digitalization, i.e., the rapid progress in and usage of information and communication technology (ICT). New fintechs changed the financial industry [1], new sharing business models such as Uber or Airbnb reshaped the taxi and hotel business, and some former incumbents even vanished, such as producers of photo film rolls [2].

The term “disruption” refers to new technologies or companies that significantly change the way that businesses operate; they wipe off older habits, competitors, or organizational systems. Whilst ICTs are now increasingly affecting and changing the healthcare sector as well, they have rarely been “disruptive” yet [3]: the major part of health care’s “business model” is still under control of the medical community; medical guidelines, for example, which define what “good quality” means, are developed and maintained by medical organizations [4]. On the other hand, ICT approaches have come to the fore and will certainly gain significantly more impact in the coming years. For example, an app–patient relationship might not only influence but as well replace the current patient–doctor relationship, and the medical profession could lose its control over the system to new market entrants. In fact, some medical specialties (like radiology and genetics) already see a transition in the distribution of power between machines and medics [5].

Thus, disruptive potential of new ICTs differs across medical specialties. In this article, we focus on rheumatology for several reasons. Many rheumatological diseases are chronic, emphasizing the process of care. In addition, many important rheumatological diseases (like, e.g., rheumatoid arthritis) are still difficult to diagnose, calling for IT support.

Focusing on rheumatology, relatively few studies have addressed the question of how new ICTs will impact health care [6, 7]. Those who did focused mainly on diagnostics and treatment: how does ICT improve medical tools and instruments? (e.g. [8–10]). In this article, we analyze an often neglected issue: the potential disruption of the management of care in rheumatology: who—person or software—takes care of the patient? Who controls the organization of health care (e.g., defines good quality and structures health care provision)? Will the balance of power between physicians, payers, politicians, and ICT companies change? And will the medical community (including its professional organizations), which currently regulates its activities autonomously, with little control from the outside, still rule the system?

As a side note, with “profession” we denote the fact that a group of professionals (like doctors or lawyers) regulate their activities on their own, with little control from the outside [11].

New ICTs are by definition new, and estimation of disruptive potential is rare. Therefore, we assumed that it is best to start with a very basic question: how many new ICTs are available in rheumatology and of what type? In a second step, we analyzed these technologies and categorized them by type so that we could tentatively analyze their disruptive potential.

## Methods

In order to identify new ICTs that change the way rheumatology is performed, we scanned the PubMed, LIVIVO, and EBSCO Discovery Service (EDS) databases for relevant literature. The following search terms were used:

- (digitalization) AND (rheumatology),
- (eHealth OR e‑health) AND (rheumatology),
- (mhealth OR mobile health OR m‑health) AND (rheumatology),
- (mhealth OR mobile health OR m‑health) AND (rheumatology OR rheumatic disease),
- (digitization OR digitalization) AND (rheumatology OR rheumatic disease),
- (ehealth OR e‑health OR telecare OR telemedicine OR telehealth) AND (rheumatology OR rheumatic disease).

Due to the nature of our research question, we did not only include clinical studies, but also nonexperimental articles. In the beginning, we considered only articles from the last 10 years, but by backward search we also included earlier articles. Thus, articles from 2008 to 2021 in German and English were considered.

We expect sociocultural and economic factors as well as the health care system to be major determinants of the impact of ICT. Therefore, we have limited our analyses to Europe and North America.

**Fig. 1:**
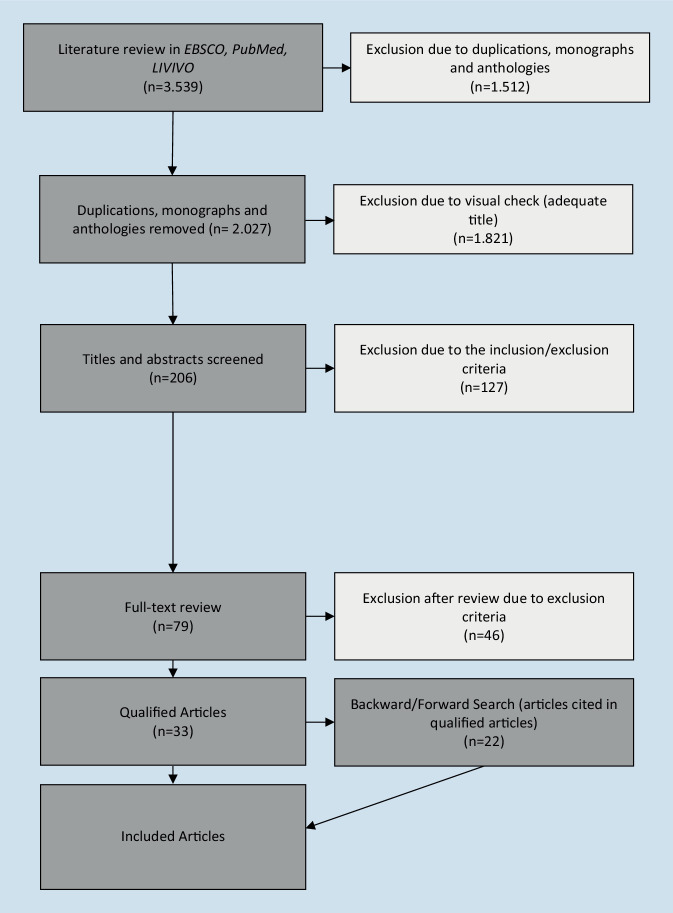
Flowchart of systematic literature review

The inclusion and exclusion criteria, as well as the search history, can be found in Fig. 1. We followed the PRISMA guidelines [12].

From the articles included, new ICTs in rheumatology were identified and extracted by content analysis (according to Mayring, modified) [13].

In a second step, we clustered these articles by their primary aim into three groups: technologies that develop new knowledge vs. those that communicate existing knowledge or change the care process. We further split the first group by the way they develop new knowledge. This resulted in four groups of ICTs:

- A.1) technologies that prepare for the development of new knowledge by data collection,
- A.2) technologies that develop new knowledge by evaluation of data (e.g., by inventing better treatment),
- B.1) technologies that improve communication of existing knowledge,
- B.2) technologies that improve the care process.

We also estimated the level of evidence of articles found. After analyzing dozens of slightly different rating systems [14], the New Zealand Guidelines Group rating system fit best with our purpose. We merged nonexperimental designs and case series into one evidence level, resulting in four levels:

1. Randomized controlled trials (RCTs),
2. Nonrandomized controlled trials,
3. Nonexperimental designs: cohort studies, case–control studies, case series, and similar, and
4. Expert opinion.

Our research did not need approval from our universities’ ethical boards.

## Results

A total of 55 relevant publications were identified. Details, including the grouping of ICTs, are provided in Table 1. A full list of retrieved articles is available as an appendix (Supplementary Table S1) to this study.

**Table 1:** Number of articles in allocated categories

Allocated categories	Number of articles
Technologies that prepare for the development of new knowledge by data collection	32
Technologies that develop new knowledge by evaluation of data (e.g., by inventing better treatment)	11
Technologies that improve communication of existing knowledge	32
Technologies that improve the care process	29

Content analysis further yielded the typical ICT approach per category.

### A.1. Technologies that prepare for the development of new knowledge by data collection (*n* = 32 studies)

Technologies in this group primarily serve the purpose of *collecting* data, aiming at the development of new knowledge. They focus on *raising* data as such—in contrary to other applications (in the group A.2) which try to generate new knowledge by *analyzing *data.

Technologies in this group serve several distinct purposes:

- Provision of new methods for data gathering from and for patients and physicians about, e.g., pain status and diaries, comorbidities, and others
- Automated data gathering (e.g., activity tracking, optical imaging of joints)
- Identification of RWE (real world evidence), that is, collecting data under real-word conditions (e.g., registries)
- Others (e.g., biobanks)

Technologies in this group can also be broken down into categories by the person who collects data:

- Collection of data by physicians or medical institutions, especially registries
- Collection of data by patients (e.g., patient-reported outcomes in electronic diaries) and their organizations
- Collection of data by others (e.g., payers or companies)

There are various parameters that are documented, e.g., physical variables, disease status or condition of the user. A validation of the data does not always take place.

Finally, these technologies differ by the type of data source they use. Whilst some try to gather data directly from the respective patient (some of them by active data entry, some of them by passive data recording), some extract data from databases (from health records up to social networks).

### A.2. Technologies that develop new knowledge by evaluation of data (e.g., by inventing better treatment; *n* = 11 studies)

A second group of technologies aims to *evaluate* data, e.g., with the help of artificial intelligence. We identified three main subgroups:

- Instruments for automated analysis of images
- ICT-supported evaluation of medical and other, e.g., administrative data (“big data”)
- Autonomous machine learning, especially in diagnosing

### B.1. Technologies that improve communication of *existing *knowledge (*n* = 32 studies)

This category is about sharing and/or exchange of existing knowledge for patients, students, physicians, and others. We identified the following technologies:

- Information for and from patients
  - Information about diseases (including enabling of shared decision-making)
  - Symptom checkers (i.e., ICT tools that diagnose)
  - Exercises for mobility, stress reduction, and rehabilitation
  - Motivational tools (e.g., nutrition, increasing physical activity)
  - Information about the “quality” of treatments and service providers
- Information for physicians and medical students
  - Diagnostic support, e.g., score calculators (for general practitioners [GPs] and/or specialists)
  - Therapy recommendations, e.g., for comorbidity or drug interaction, and guidelines
  - Access to medical knowledge, e.g., through online libraries
  - Educational tools (e.g., virtual reality)
- Data exchange/data cooperation
  - Communication (including data exchange) between patients
  - Communication between doctors, including primary care and specialists (e.g., virtual consultation)
  - Data and information exchange between patients and physicians/hospitals; video consultation
  - Other data exchange (e.g., between patients and insurance companies)

### B.2. Process-oriented applications that improve the care process (*n* = 29 studies)

This category includes technologies that support or (co-)control the process in medicine, e.g., the coordination of doctor’s appointments, but also patient flow.

- Coordination of doctor’s appointments, reminder function (medication, appointments, documentation)
- Management of disease documentation (e.g., integration of clinical documentation into hospital ICT systems)
- Automated screening and detection of unknown disease and/or progress of disease in order to identify the need for consultation
- Automated triage (that is, estimation of the need for specialist care)
- New ICT infrastructure for data exchange between physicians and others; with or without access to electronic health records

Obviously, there is some overlap between purposes: for example, a technology that gathers data from a patient may sometimes be more focused on the data as such (e.g., for scientific research), on the communication of these data to a physician (so that there is additional data available), or on the process of data transfer as such (e.g., the patient enters data that the doctor would otherwise inquire).

We found the following levels of evidence (Table 2).

**Table 2:** Number of studies in the respective level

Level	Number of studies
Randomized controlled trials	2
Nonrandomized controlled trials	5
Nonexperimental designs	31
Expert opinion	17

## Discussion

In its recent report about ethics and governance of artificial intelligence for health, the World Health Organization warns: “… if we do not take appropriate measures, AI could … lead to situations where decisions that should be made by providers and patients are transferred to machines, which would undermine human autonomy, as humans may neither understand how an AI technology arrives at a decision, nor be able to negotiate with a technology to reach a shared decision.” [15].

Given the importance of new ICT developments and their disruptive potential for medicine, and rheumatology in particular, the 55 articles that met our inclusion criteria seem to be a relatively small number.

The few articles we found deal with very different issues, ranging from data collection and evaluation, telemedicine, artificial intelligence, wearables, apps, remote monitoring over communication to process management—that is, scientific evidence is not only scarce but also spread over a variety of issues. We conclude that there is urgent need for more research into the question of how ICT will influence patient management in rheumatology, especially about the patient–doctor relationship, the medical profession, and the organization of treatment.

The medical profession is currently focused on researching the potential of ICT to improve diagnosis and therapy and, sometimes, process management [16, 17]. We recommend taking a view on the governance side of medicine as well.

This holds true even more since there is a boom of political reforms to establish several digital tools (such as video-based doctor consulting and provision of electronic prescriptions). In Germany, this is called the “e-health initiative” [18]; e.g., statutory health insurances have to pay for certain health apps [19]. At the same time, not all doctors seem to be ready for digitization. A survey in February 2021 revealed a lack of digital competence with the use of digital health apps. This barrier was named as reason that only one quarter of doctors are willing to prescribe digital health apps to their patients [20]. This will also make it difficult for doctors to keep a sound grasp of new ICT developments. For rheumatology, a recent survey found that only a minority of German physicians use ePROs (electronic patient-reported outcomes), and the main reason for not implementing them was cited as the unawareness of suitable software solutions [21].

It is apparent that there is a multitude of varying approaches in rheumatological ICT which differ not only in purpose but also in disruptive potential. Categorization appears useful in order to be able to classify the degrees of impact of the various applications on different areas, such as treatment, research, development, and others.

The four ICT groups we identified may help to establish a first, tentative estimate of the disruptive potential of new technologies:

1. Group A1 focuses on the development of new knowledge by data collection. As long as these data are accessible and understandable for everybody, we don’t see much disruptive potential. This might differ if, for some reason, some participants keep data secretly and gain proprietary medical knowledge. It also depends on the type of data gathered and who interprets them (e.g., doctors or machines). Up to today, large amounts of patient data are still not used as “big data.” Rather, data are collected by insurers and various other stakeholders/companies acting in the health care market (although data are, originally, created by physicians). For example, in 2014, a mid-sized company already had access to 85% of global prescriptions by sales revenue and approximately 400 million comprehensive, longitudinal patient records [22]. Of course, big IT companies with more financial power could even buy better data access. For example, Apple (Cupertino, CA, USA) recently became the first company to exceed $ 3 trillion market value [23]; this compares well to German yearly GDP (the value of all products and services produced) which is about €3.5 trillion [24].
2. The same estimation of disruptive potential holds true for technologies that develop new knowledge by evaluation of data (e.g., by inventing better treatment)—group A2: as long as these insights are publicly available and the medical profession keeps control over them, there will be little disruption. On the other hand, if non-public companies collect and analyze data, develop a better understanding of diagnostic and therapeutic procedures, and keep this knowledge for themselves, they could gain the power to define good quality in medicine. This phenomenon could happen when self-learning algorithms determine treatment decisions without any control by real doctors.
  Another potential change is that human beings—including doctors—are unable to understand and interpret ICT recommendations anymore (the “black box problem,” which is the case in chess already: even the best human chess players sometimes do not understand *why* software “thinks” a certain move is the best).
  Disruptive potential was mitigated if machine learning is interpretable—that is, humans can understand the decisions made by machines.
  Ultimately, this means that data evaluation technologies will not so much change the patient–physician relationship (as long as they are not combined with technologies in B.1, such as artificial nurses and doctors which communicate with patients), but could disrupt the medical profession—because the profession could lose the control over the definition of “good quality.” This will be all the more the case if collected raw data become invisible for patient and physician but rather proprietary knowledge of some third party.
3. Technologies that improve communication of existing knowledge (group B1) might interfere with current practice if they bypass the patient–doctor relationship. Especially if combined with technologies from groups A1 and A2 (which collect and evaluate data), the provision of medical care could change massively.
4. Technologies that improve the care process (B2) will disrupt only if they bring along a new way of control over the care process.

This also holds true for combinations of these technological groups. Combining superior knowledge with tools for communication with patients (e.g., artificial, AI-based agents) could theoretically support doctors but also make them obsolete at critical treatment steps.

In essence, ICT as such is not the key to disruption, as long as it doesn’t change *the management of care in rheumatology*. In other words, the key question is whether new ICTs are merely new instruments for the benefit of patients and in the hand of doctors, or if they bypass or take control over quality measurement, physicians, and patients. This is also in line with earlier European Alliance of Associations for Rheumatology (EULAR) recommendations. For example, EULAR focused on patient benefits in patient self-management apps [25] but mentioned potential risks in big data use [26].

New technologies such as better data gathering, analysis, and communication are both powerful and useful instruments for care improvement as long as patients and physicians have access to this information; if they are “locked out,” changes may be both disruptive as well as harmful. Also, they may be risky if quality control of new ICTs is not as rigid as in, e.g., drug development.

Digitalization has the potential to significantly improve the quality of health care. However, many industries have seen disruption so far, some of which may harm consumers and/or vendors. Rheumatology is in no way sheltered from the disruptive potential of new ICT technologies. The topic has hardly been researched so far. We found that only few of the articles retrieved were RCTs; it may be that disruptive developments are difficult to detail in RCTs, and research in this area does not fit well to typical medical journals.

An important result of this study is that these developments should be closely monitored and accompanied by patient representatives, medical institutions (such as chambers of physicians), scientific medical societies, health insurances and other relevant stakeholders. For example, the EULAR recommendations for drugs [27] could be helpful and extended to ICT and governance, safeguarding benefits for patient as well as security standards.

## Supplementary Information

Tab. S1 Articles included in the study

## Declarations

For this article no studies with human participants or animals were performed by any of the authors.
