# Supplementary material for: Digitalization and disruptive change in rheumatology
Source: Z Rheumatol. 2022 May 31;82(5):417–24. doi: 10.1007/s00393-022-01222-4 (PMC10264504; doi:10.1007/s00393-022-01222-4)
Supplement: Supplementary file 1 — Tab. S1 Articles included in the study [file 393_2022_1222_MOESM1_ESM.pdf]

Tab. S1 Articles included

Source	A1	A2	B1	B2	Level of evidence
Albrecht K, Huscher D, Eidner T, Kleinert S, Späthling-Mestekemper S, Bischoff S, Zink A. Versorgung der rheumatoiden Arthritis 2014 : Aktuelle Daten aus der Kerndokumentation [Medical treatment of rheumatoid arthritis in 2014 : Current data from the German Collaborative Arthritis Centers]. Z Rheumatol. 2017 Feb;76(1):50-57. German. doi: 10.1007/s00393-016-0156-5. PMID: 27379740	x	x			3
Aries P, Welcker M, Callhoff J, Chehab G, Krusche M, Schneider M, Specker C, Richter JG. Stellungnahme der Deutschen Gesellschaft für Rheumatologie e. V. (DGRh) zur Anwendung der Videosprechstunde in der Rheumatologie [Statement of the German Society for Rheumatology (DGRh) on the use of video consultations in rheumatology]. Z Rheumatol. 2020 Dec;79(10):1078-1085. German. doi: 10.1007/s00393-020-00932-x. Epub 2020 Nov 17. PMID: 33201305; PMCID: PMC7670291.				x	4
Azevedo R, Bernardes M, Fonseca J, Lima A. Smartphone application for rheumatoid arthritis self-management: cross-sectional study revealed the usefulness, willingness to use and patients' needs. Rheumatol Int. 2015 Oct;35(10):1675-85. doi: 10.1007/s00296-015-3270-9. Epub 2015 Apr 24. PMID: 25903352	x		x	x	3
Burmester GR, Häupl T. „Big Data" – auch in der Rheumatologie relevant? [Big data-also relevant in rheumatology?]. Z Rheumatol. 2018 Apr;77(3):192-194. German. doi: 10.1007/s00393-018-0437-2. PMID: 29619762	x	x			4
Burmester GR. Rheumatology 4.0: big data, wearables and diagnosis by computer. Ann Rheum Dis. 2018 Jul;77(7):963-965. doi: 10.1136/annrheumdis-2017-212888. Epub 2018 May 25. PMID: 29802224; PMCID: PMC6029631	x	x			4
Catarinella FS, Bos WH. Digital health assessment in rheumatology: current and future possibilities. Clin Exp Rheumatol. 2016 Sep-Oct;34(5 Suppl 101):S2-S4. Epub 2016 Oct 18. PMID: 27762198	x			x	4
Davergne T, Rakotozafiarison A, Servy H, Gossec L. Wearable Activity Trackers in the Management of Rheumatic Diseases: Where Are We in 2020? Sensors (Basel). 2020 Aug 25;20(17):4797. doi: 10.3390/s20174797. PMID: 32854412; PMCID: PMC7506912	x				4
DiBonaventura M, Pomerantz D, Mondry B: PMS64 - The real-world pain experience of adults with rheumatoid arthritis using a mobile survey. Value in Health 2016; 19 (3): A236-A236	x				3
Dixon WG, Michaud K. Using technology to support clinical care and research in rheumatoid arthritis. Curr Opin Rheumatol. 2018 May;30(3):276-281. doi: 10.1097/BOR.0000000000000485. PMID: 29369089; PMCID: PMC5895111	x			x	4

El Miedany Y. e-Rheumatology: are we ready? Clin Rheumatol. 2015 May;34(5):831-7. doi: 10.1007/s10067-015-2897-y. Epub 2015 Feb 25. PMID: 25708153	x	x	x	x	4
Gossec L, Guyard F, Leroy D, Lafargue T, Seiler M, Jacquemin C, Molto A, Sellam J, Foltz V, Gandjbakhch F, Hudry C, Mitrovic S, Fautrel B, Servy H. Detection of Flares by Decrease in Physical Activity, Collected Using Wearable Activity Trackers in Rheumatoid Arthritis or Axial Spondyloarthritis: An Application of Machine Learning Analyses in Rheumatology. Arthritis Care Res (Hoboken). 2019 Oct;71(10):1336-1343. doi: 10.1002/acr.23768. PMID: 30242992	x	x			3
Ho K, Yao C, Novak Lauscher H, Koehler BE, Shojania K, Jamal S, Collins D, Kherani R, Meneilly G, Eva K. Remote assessment via video evaluation (RAVVE): a pilot study to trial video-enabled peer feedback on clinical performance. BMC Med Educ. 2019 Dec 18;19(1):466. doi: 10.1186/s12909-019-1905-3. PMID: 31852496; PMCID: PMC6921528			x		3
Hoogland J, Wijnen A, Munsterman T, Gerritsma CL, Dijkstra B, Zijlstra WP, Annegarn J, Ibarra F, Zijlstra W, Stevens M. Feasibility and Patient Experience of a Home-Based Rehabilitation Program Driven by a Tablet App and Mobility Monitoring for Patients After a Total Hip Arthroplasty. JMIR Mhealth Uhealth. 2019 Jan 31;7(1):e10342. doi: 10.2196/10342. PMID: 30702438; PMCID: PMC6374724			x		3
Howren A, Tsao NW, Choi HK, Shojania K, Kydd A, Friesen R, Avina-Zubieta JA, De Vera MA. eHealth-supported decentralized multi-disciplinary care for gout involving rheumatology, pharmacy, and dietetics: proof-of-concept study. Clin Rheumatol. 2020 Apr;39(4):1241-1249. doi: 10.1007/s10067-019-04809-6. Epub 2019 Nov 13. PMID: 31720913			x		3
Hummel, M., Illig, T., Jahns, R.: Aktuelle Herausforderungen und Chancen im Biobanking, Tagungsband des 6. Nationalen Biobanken-Symposiums vom 6.-7. Dezember 2017 in Berlin	x				4
Kataria S, Ravindran V. Digital health: a new dimension in rheumatology patient care. Rheumatol Int. 2018 Nov;38(11):1949-1957. doi: 10.1007/s00296-018-4037-x. Epub 2018 Apr 30. PMID: 29713795	x	x	x	x	4
Kiltz U, Boonen A, Braun J, Richter JG. Electronic assessment of disease activity and functioning in patients with axial spondyloarthritis: challenges and unmet needs. Clin Exp Rheumatol. 2016 Sep-Oct;34(5 Suppl 101):S57-S61. Epub 2016 Oct 18. PMID: 27762203	x			x	3
Kleyer A, Simon D, Hartmann F, Schuster L, Hueber AJ. „Virtuelle Rheumatologie“ : Ein neues Lehrkonzept für die Rheumatologie der Zukunft? ["Virtual rheumatology" : A new teaching concept for rheumatology of the future?]. Z Rheumatol. 2019 Mar;78(2):112-115. German. doi: 10.1007/s00393-019-0594-y. PMID: 30694358			x		4

Knitza J, Kleyer A, Klüppel M, Krauser M, Wacker J, Schett G, Simon D. Online-Ultraschalllernmodule in der Rheumatologie : Innovatives Wahlfach steigert studentisches Interesse für das Fach Rheumatologie [Online ultrasound learning modules in rheumatology : Innovative elective course increases student interest in the discipline of rheumatology]. Z Rheumatol. 2020 Apr;79(3):276-279. German. doi: 10.1007/s00393-020-00757-8. PMID: 32103342			x		3
Knitza J, Knevel R, Raza K, Bruce T, Eimer E, Gehring I, Mathsson-Alm L, Poorafshar M, Hueber AJ, Schett G, Johannesson M, Catrina A, Klareskog L; JPAST Group. Toward Earlier Diagnosis Using Combined eHealth Tools in Rheumatology: The Joint Pain Assessment Scoring Tool (JPAST) Project. JMIR Mhealth Uhealth. 2020 May 15;8(5):e17507. doi: 10.2196/17507. PMID: 32348258; PMCID: PMC7260666			x	x	3
Knitza J, Simon D, Lambrecht A, Raab C, Tascilar K, Hagen M, Kleyer A, Bayat S, Derungs A, Amft O, Schett G, Hueber AJ. Mobile Health Usage, Preferences, Barriers, and eHealth Literacy in Rheumatology: Patient Survey Study. JMIR Mhealth Uhealth. 2020 Aug 12;8(8):e19661. doi: 10.2196/19661. PMID: 32678796; PMCID: PMC7450373	x		x	x	3
Knitza J, Tascilar K, Messner EM, Meyer M, Vossen D, Pulla A, Bosch P, Kittler J, Kleyer A, Sewerin P, Mucke J, Haase I, Simon D, Krusche M. German Mobile Apps in Rheumatology: Review and Analysis Using the Mobile Application Rating Scale (MARS). JMIR Mhealth Uhealth. 2019 Aug 5;7(8):e14991. doi: 10.2196/14991. PMID: 31381501; PMCID: PMC6699116	x		x	x	3
Knitza J, Vossen D, Geffken I, Krusche M, Meyer M, Sewerin P, Kleyer A, Hueber AJ; Arbeitskreis Junge Rheumatologen. Nutzung von Medizin-Apps und Online-Plattformen unter deutschen Rheumatologen : Ergebnisse der rheumadocs-Recherche und DGRh-Kongress-Umfragen von 2016 und 2018 [Use of medical apps and online platforms among German rheumatologists : Results of the 2016 and 2018 DGRh conference surveys and research conducted by rheumadocs]. Z Rheumatol. 2019 Nov;78(9):839-846. German. doi: 10.1007/s00393-018-0578-3. PMID: 30542914			x		3
Krusche M, Mühlensiepen F, Aries P, Welcker M, Knitza J. Telemedizin in der Rheumatologie [Telemedicine in rheumatology]. Z Rheumatol. 2020 Nov;79(9):883-892. German. doi: 10.1007/s00393-020-00912-1. Epub 2020 Oct 15. PMID: 33057786; PMCID: PMC7557255			x	x	4
Krusche M, Ruffer N, Grahammer M, Knitza J. Apps und ihre Anwendungsgebiete in der Rheumatologie [Mobile apps and their usage in rheumatology]. Z Rheumatol. 2020 Aug;79(6):554-561. German. doi: 10.1007/s00393-020-00822-2. PMID: 32472178	x		x	x	4
Maurits, M., Yuminaga, H., Huizinga, et al.: Mobile health applications in rheumatology: Could they improve our care and research?, in: International journal of clinical rheumatology, 14 (2019), Nr. 1:44–49	x	x	x	x	4

Mollard E, Michaud K. A Mobile App With Optical Imaging for the Self-Management of Hand Rheumatoid Arthritis: Pilot Study. JMIR Mhealth Uhealth. 2018 Oct 29;6(10):e12221. doi: 10.2196/12221. PMID: 30373732; PMCID: PMC6234331	x			x	2
Mucke J, Sewerin P, Schneider M. Rheumatology in 2049: the age of all data. Ann Rheum Dis. 2021 Jul;80(7):825-827. doi: 10.1136/annrheumdis-2020-219686. Epub 2021 Feb 9. PMID: 33563594		x		x	4
Mühlensiepen F, Kurkowski S, Krusche M, Mucke J, Prill R, Heinze M, Welcker M, Schulze-Koops H, Vuillerme N, Schett G, Knitza J. Digital Health Transition in Rheumatology: A Qualitative Study. Int J Environ Res Public Health. 2021 Mar 5;18(5):2636. doi: 10.3390/ijerph18052636. PMID: 33807952; PMCID: PMC7967307	x	x	x		3
Mühlensiepen F, Marquardt W, Welcker M. Machbarkeitsstudie: Implementierung eines telemedizinischen Versorgungskonzeptes in die Rheumatologie im Land Brandenburg (TeleRheumaBB) – Zwischenergebnisse der Fragebogenerhebung, Deutsches Netzwerk Versorgungsforschung, 2019, doi: 10.3205/19DKVF064			x	x	3
Powley L, McIlroy G, Simons G, Raza K. Are online symptoms checkers useful for patients with inflammatory arthritis? BMC Musculoskelet Disord. 2016 Aug 24;17(1):362. doi: 10.1186/s12891-016-1189-2. PMID: 27553253; PMCID: PMC4995741			x		2
Proft F, Spiller L, Protopopov M, et al. THU0230 Performance of an online self-referral questionnaire compared to a physician-based referral approach to identify patients with a high probability of axial spondyloarthritis: results from the optiref study. Annals of the Rheumatic Diseases 2018;77:335			x	x	3
Prvu Bettger J, Green CL, Holmes DN, Chokshi A, Mather RC 3rd, Hoch BT, de Leon AJ, Aluisio F, Seyler TM, Del Gaizo DJ, Chiavetta J, Webb L, Miller V, Smith JM, Peterson ED. Effects of Virtual Exercise Rehabilitation In-Home Therapy Compared with Traditional Care After Total Knee Arthroplasty: VERITAS, a Randomized Controlled Trial. J Bone Joint Surg Am. 2020 Jan 15;102(2):101-109. doi: 10.2106/JBJS.19.00695. PMID: 31743238			x		2
Revenäs Å, Opava CH, Ahlén H, Brusewitz M, Pettersson S, Åsenlöf P. Mobile internet service for self-management of physical activity in people with rheumatoid arthritis: evaluation of a test version. RMD Open. 2016 Apr 4;2(1):e000214. doi: 10.1136/rmdopen-2015-000214. PMID: 27099777; PMCID: PMC4823585	x		x		3
Richter JG, Becker A, Koch T, Nixdorf M, Willers R, Monser R, Schacher B, Alten R, Specker C, Schneider M. Self-assessments of patients via Tablet PC in routine patient care: comparison with standardised paper questionnaires. Ann Rheum Dis. 2008 Dec;67(12):1739-41. doi: 10.1136/ard.2008.090209. Epub 2008 Jul 22. PMID: 18647853	x				3

Richter JG, Becker A, Schalis H, Koch T, Willers R, Specker C, Monser R, Schneider M. An ask-the-expert service on a rheumatology web site: who were the users and what did they look for? Arthritis Care Res (Hoboken). 2011 Apr;63(4):604-11. doi: 10.1002/acr.20399. PMID: 21452271			x		3
Richter J, Bleck E, Acar H. et al. Sicheres Biobanking bei entzündlich-rheumatischen Erkrankungen: erste deutsche Erfahrungen. In: Hummel M, Illig T, Jahns R. et al. Hrsg. Aktuelle Herausforderungen und Chancen im Biobanking. 6. Nationales Biobanken-Symposium. Tagungsband. Berlin: 2017: 171-173	x				4
Richter JG, Chehab G, Kiltz U, Callhoff J, Voormann A, Lorenz HM, Schneider M, Specker C. Digital Health in der Rheumatologie – Statuserhebung 2018/19 [Digital Health in Rheumatology - Status 2018/19]. Dtsch Med Wochenschr. 2019 Apr;144(7):464-469. German. doi: 10.1055/a-0740-8773. Epub 2019 Mar 29. Erratum in: Dtsch Med Wochenschr. 2019 Apr;144(7):e3. PMID: 30925601	x	x	x	x	4
Richter JG, Chehab G, Schneider M. Electronic health records in rheumatology: emphasis on automated scoring and additional use. Clin Exp Rheumatol. 2016 Sep-Oct;34(5 Suppl 101):S62-S68. Epub 2016 Oct 18. PMID: 27762191	x			x	3
Richter JG, Chehab G, Schneider M. THU0625 Design of an information and communications technology platform to support coordination of care for rheumatoid arthritis patients with cardiovascular co-morbidities – first experiences. Annals of the Rheumatic Diseases. 2017; 76: 441-442			x		3
Richter JG, Kampling C, Chehab G. et al. Patient-reported outcome Instrumente bei Rheumatoider Arthritis – liefert die RheumaLive App valide Daten? Meeting abstract, 44. Kongress der Deutschen Gesellschaft für Rheumatologie, 30. Jahrestagung der Deutschen Gesellschaft für Orthopädische Rheumatologie, 26. Jahrestagung der Gesellschaft für Kinder- und Jugendrheumatologie, 2016, doi: 10.3205/16dgrh032	x		x	x	2
Richter JG, Wessel E, Klimt R, Willers R, Schneider M. RheumaCheck: Entwicklung und Evaluation eines deutschsprachigen Rheuma-Screening Instruments [RheumaCheck: development and evaluation of a German language screening instrument for rheumatic diseases]. Wien Klin Wochenschr. 2008;120(3-4):103-11. German. doi: 10.1007/s00508-008-0929-1. PMID: 18322772	x			x	2
Russell TG, Buttrum P, Wootton R, Jull GA. Internet-based outpatient telerehabilitation for patients following total knee arthroplasty: a randomized controlled trial. J Bone Joint Surg Am. 2011 Jan 19;93(2):113-20. doi: 10.2106/JBJS.I.01375. PMID: 21248209			x		1
Salaffi F, Carotti M, Ciapetti A, Di Carlo M, Gasparini S, Farah S, Gutierrez M. Effectiveness of a telemonitoring intensive strategy in early rheumatoid arthritis: comparison with the conventional management approach. BMC Musculoskelet Disord. 2016 Apr 2;17:146. doi: 10.1186/s12891-016-1002-2. PMID: 27038788; PMCID: PMC4818962	x			x	1

Sapir T, Rusie E, Greene L, Yazdany J, Robbins ML, Ruderman EM, Carter JD, Patel B, Moreo K. Influence of Continuing Medical Education on Rheumatologists' Performance on National Quality Measures for Rheumatoid Arthritis. Rheumatol Ther. 2015 Dec;2(2):141-151. doi: 10.1007/s40744-015-0018-7. Epub 2015 Oct 1. PMID: 27747535; PMCID: PMC4883265			x		3
Schwarting A, Dreher M, Assmann G, Witte T, Hoeper K, Schmidt RE. Erfahrungen und Ergebnisse aus Rheuma-VOR [Experiences and results from Rheuma-VOR]. Z Rheumatol. 2019 Oct;78(8):743-752. German. doi: 10.1007/s00393-019-00694-1. PMID: 31468168				x	3
Schwarting A, Pfeiff B, Amberger C, Pick D, Hesse M, Jendro M, Engels J, Böttger A, Kuhn C, Majdandzic J, Ziese W, Stadelmann ML, Kessler FW, Dinges H, Ultes-Kaiser S, Droste U, Schmalhofer M, Hazenbiller A, Rector M, Weinmann-Menke J, Triantafyllias K, Becker M, Ataian M, Lablans M, Ueckert F, Panholzer T, Blettner M. Das landesweite Netzwerk ADAPTHERA : Erste Ergebnisse einer flächendeckenden, krankenkassenübergreifenden und transsektoralen rheumatologischen Versorgung [The regional network ADAPTHERA : Rheumatology care through coordinated cooperation: comprehensive, trans-sectoral, covering all health insurance. Initial results]. Z Rheumatol. 2016 Dec;75(10):999-1005. German. doi: 10.1007/s00393-016-0132-0. PMID: 27535273	x			x	3
Seppen BF, Wiegel J, L'ami MJ, Duarte Dos Santos Rico S, Catarinella FS, Turkstra F, Boers M, Bos WH. Feasibility of Self-Monitoring Rheumatoid Arthritis With a Smartphone App: Results of Two Mixed-Methods Pilot Studies. JMIR Form Res. 2020 Sep 21;4(9):e20165. doi: 10.2196/20165. PMID: 32955447; PMCID: PMC7536594	x		x	x	3
Smarr KL, Musser DR, Shigaki CL, Johnson R, Hanson KD, Siva C. Online self-management in rheumatoid arthritis: a patient-centered model application. Telemed J E Health. 2011 Mar;17(2):104-10. doi: 10.1089/tmj.2010.0116. Epub 2011 Mar 1. PMID: 21361817	x		x	x	3
Sunkureddi P, Gibson D, Doogan S, Heid J, Benosman S, Park Y. Using Self-Reported Patient Experiences to Understand Patient Burden: Learnings from Digital Patient Communities in Ankylosing Spondylitis. Adv Ther. 2018 Mar;35(3):424-437. doi: 10.1007/s12325-018-0669-1. Epub 2018 Feb 15. PMID: 29450863; PMCID: PMC5859700	x				3
Tejera Segura B, Bustabad S. A new form of communication between rheumatology and primary care: The virtual consultation. Reumatol Clin. 2016 Jan-Feb;12(1):11-4. English, Spanish. doi: 10.1016/j.reuma.2015.01.003. Epub 2015 Feb 14. PMID: 25687555			x	x	3
van Riel PLCM, Zuidema RM, Vogel C, Rongen-van Dartel SAA. Patient Self-Management and Tracking: A European Experience. Rheum Dis Clin North Am. 2019 May;45(2):187-195. doi: 10.1016/j.rdc.2019.01.008. PMID: 30952392	x		x		3

Ward IM, Schmidt TW, Lappan C, Battafarano DF. How Critical is Tele-Medicine to the Rheumatology Workforce? Arthritis Care Res (Hoboken). 2016 Oct;68(10):1387-9. doi: 10.1002/acr.22853. Epub 2016 Aug 19. PMID: 26866514			x	x	4
Wilkinson MD, Dumontier M, Aalbersberg IJ, Appleton G, Axton M, Baak A, Blomberg N, Boiten JW, da Silva Santos LB, Bourne PE, Bouwman J, Brookes AJ, Clark T, Crosas M, Dillo I, Dumon O, Edmunds S, Evelo CT, Finkers R, Gonzalez-Beltran A, Gray AJ, Groth P, Goble C, Grethe JS, Heringa J, 't Hoen PA, Hooft R, Kuhn T, Kok R, Kok J, Lusher SJ, Martone ME, Mons A, Packer AL, Persson B, Rocca-Serra P, Roos M, van Schaik R, Sansone SA, Schultes E, Sengstag T, Slater T, Strawn G, Swertz MA, Thompson M, van der Lei J, van Mulligen E, Velterop J, Waagmeester A, Wittenburg P, Wolstencroft K, Zhao J, Mons B. The FAIR Guiding Principles for scientific data management and stewardship. Sci Data. 2016 Mar 15;3:160018. doi: 10.1038/sdata.2016.18. Erratum in: Sci Data. 2019 Mar 19;6(1):6. PMID: 26978244; PMCID: PMC4792175		x			3
Wojnowski L, Dreher M, Hilt A. et al.: Die Rheuma-VOR App: Optimierung der Frühdiagnose von rheumatischen Erkrankungen, 2019, Nr. GMS I 18. Deutscher Kongress für Versorgungsforschung I Meeting Abstract				x	3
